# Supplementary material for: Recruiting and Engaging American Indian and Alaska Native Teens and Young Adults in a SMS Help-Seeking Intervention: Lessons Learned from the BRAVE Study
Source: Int J Environ Res Public Health. 2020 Dec 16;17(24):9437. doi: 10.3390/ijerph17249437 (PMC7765783; doi:10.3390/ijerph17249437)
Supplement: Supplementary file 1 [file ijerph-17-09437-s001.zip › Supplementary/Supplement 1.pdf]

Brave Role Model Video Content and Links

- Alex (played by Dyami Thomas) is a 23-year-old young man born and raised on the Rez. His mom works for the Indian Health Service; his dad is barely around. He was a star basketball player in high school, was offered several college scholarships, but decided to stay home. He started drinking to celebrate his team's wins and cope with their losses. When Alex's drinking starts to spiral out of control, he gets support and guidance from a Counselor.
- Chris (played by Tashoni Morales) is a 22-year-old young woman raised by her Grandmother in a border town. Her Grandmother lives a simple life. She goes to church, follows the rules, works hard, and was raised to be proud of who she is – a strong Native woman. Chris is driven to become a doctor, to give back to her people. When her relationship turns violent, she gets support from her Aunties.
- Benny (played by Keenan Carolin) is a 23-year-old young man pushed to be successful by his father who is an active member on the Tribal Economic Development Council. In High School Benny started making videos on the Rez and quickly gained a large following on social media. When he becomes worried about his friends, Alex and Chris, he reaches out and gets support from the National Suicide Prevention Lifeline.

BRAVE and Control Arm Messages

**Tuesday**<br><br>We R Native: Many of us have grown up in traumatic environments. Now it's up to us to break the cycle. Having people in our lives who love and care about us is one way to build resilience.<br><br>**Week 2 Challenge:** Reach out to a friend or family member this week. Let them know you appreciate them and their support.<br><br>SUPPORT SYSTEM IMAGE	**Tuesday**<br><br>STEM: The American Indian College Fund has one unwavering purpose – the increase the number of American Indians who hold college degrees. Currently only 14% of American Indians have a college degree – less than half the national average. Every year, they empower more than 4,000 AI/AN students to start and stay in school, and they intend to double their impact in the next five years: https://collegefund.org/student-resources/<br><br>THINK INDIAN IMAGE
**Thursday**<br><br>We R Native: Alex's drinking was out of control. Drinking in moderation is defined as having no more than 1 drink per day for women and no more than 2 drinks per day for men. Drinking too much can cause lots of negative outcomes. Text **MORE** to learn more.<br><br>**MORE**: Regular excessive alcohol use is associated with hangovers, reduced sexual performance, aggressive behavior, accidents and injury, anxiety and depression, relationship difficulties, and suicide... just to name a few. Drinking in moderation (or not drinking at all) is the best way to keep in control.	**Thursday**<br><br>STEM: Would you like free, online courses on coding or programming? Here are a bunch to get you started: http://lil.ms/1vha

BRAVE Help-seeking Role Model Videos

- Recruitment Trailer: https://www.youtube.com/watch?v=hSAnVwlT7uk&feature=youtu.be
- Episode 1- Alex: https://youtu.be/dOcthWY9CLI
- Episode 2- Chris: https://youtu.be/9aRxIQd_62E
- Episode 3- Benny: https://youtu.be/k3YycAzDYkc
- Episode 4- Alex: https://youtu.be/SfaJBE7ttdk
- Episode 5- Chris: https://youtu.be/VpW035EXdQM
- Episode 6- Benny: https://youtu.be/f8EubVYcrB0
- Episode 7- Homecoming: https://youtu.be/_RwwkiuMNHw
- Full-length video: https://www.youtube.com/playlist?list=PLvLfi7yZ2zQHLab-9rhDqKwt5pn5bPKzu

Enrollment Messages

> Thanks for joining our study! Helping your friends and striving to grow takes courage. *We R Native* is here for you. Your texts are always PRIVATE & CONFIDENTIAL. Survey questions coming your way starting tomorrow. If you answer all of the questions you'll get a $10 amazon gift code at the end!

Those who expressed interest received up to three reminders to complete the pre-survey:

> *We R Native*: Hi there, it's important to us to learn whether these messages are helpful to you. We'll send you survey questions at the beginning, end, and at 4 and 7-8 months. Here are the first set of questions. Be sure to answer all of them to earn $10. Your answers are PRIVATE. Start the survey at http://lil.ms/1v5x
