# Supplementary material for: Recruiting and Engaging American Indian and Alaska Native Teens and Young Adults in a SMS Help-Seeking Intervention: Lessons Learned from the BRAVE Study
Source: Int J Environ Res Public Health. 2020 Dec 16;17(24):9437. doi: 10.3390/ijerph17249437 (PMC7765783; doi:10.3390/ijerph17249437)
Supplement: Supplementary file 1 [file ijerph-17-09437-s001.zip › Supplementary/Supplement 2.pdf]

Figures Supplement 2

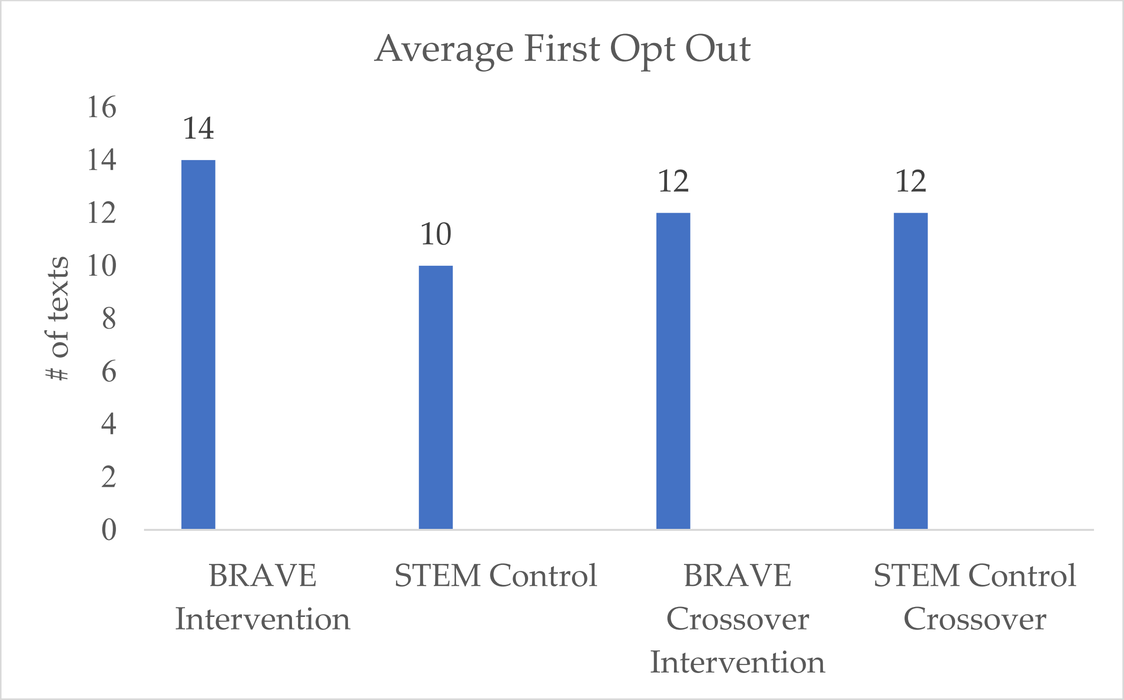


**Figure 1.** This is a figure describing the BRAVE and STEM opt-outs based on the number of text received.

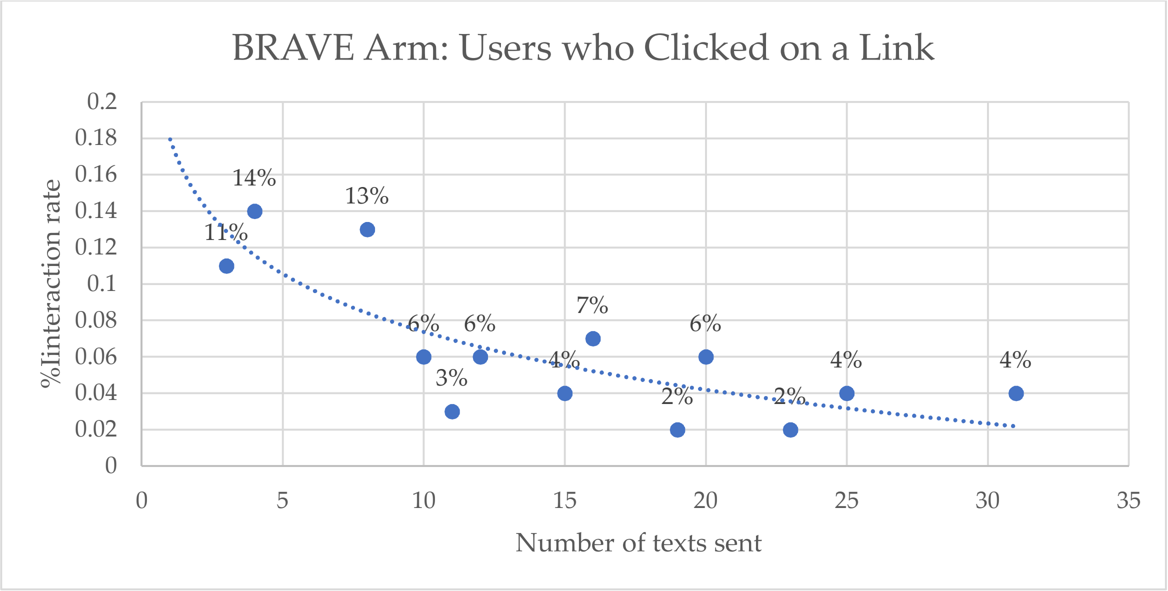


**Figure 2.** This is a figure showing the BRAVE arm intervention users interaction rate and text messages sent.

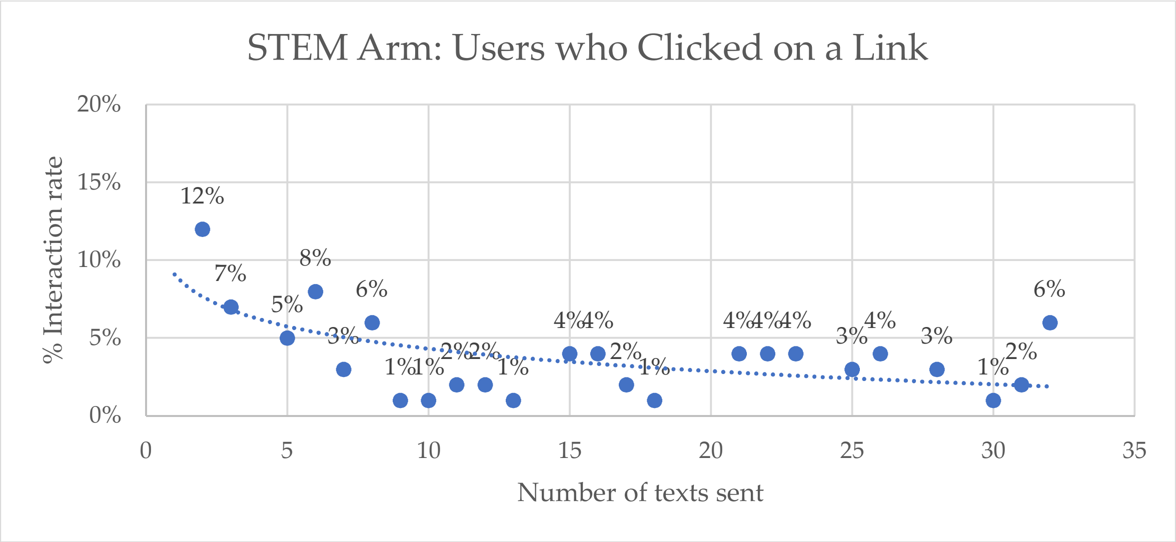


**Figure 3.** This is a figure showing the STEM arm control group interaction rate and text messages sent.
